# Adult esophageal foreign body and food impaction: a video-based guide to endoscopic and nonendoscopic management

**DOI:** 10.1016/j.igie.2026.01.002

**Published:** 2026-01-21

**Authors:** Ammar Ismail, Amira Alnakeb, Ibrahim Elhaj, Hassan Siddiki

**Affiliations:** Department of Gastroenterology, Digestive Disease and Surgery Institute, Cleveland Clinic, Cleveland, Ohio, USA

## Abstract

Esophageal foreign body (FB) ingestion and food impaction represent critical and increasingly common emergencies in adult populations, often requiring prompt diagnosis and intervention to prevent severe adverse events. This review explores the epidemiology, risk factors, and clinical management of esophageal FB and food bolus impactions, with an emphasis on endoscopic retrieval strategies. Although conservative and pharmacologic treatments, including glucagon, effervescent agents, and muscle relaxants, are occasionally used, they exhibit limited efficacy and should not delay endoscopic intervention. Flexible endoscopy remains the reference standard for diagnosis and treatment. We also discuss the use of various endoscopic tools and adjunctive devices, as well as esophageal dilation techniques for the management of esophageal impaction and underlying pathologies. This review aims to guide clinicians in approaching cases with esophageal impaction and in selecting the most effective and safest endoscopic approaches for managing adult esophageal impactions. A supplementary video demonstrates key retrieval tools in a simulated setting.

## Background

Esophageal foreign body impaction (EFBI) and food bolus impaction represent urgent clinical conditions frequently encountered in both pediatric and adult populations. Although children account for the majority of cases, recent years have seen a noticeable rise in incidence among adults.^1^ Eosinophilic esophagitis (EoE) has emerged as a leading contributor, particularly in younger adults and those with allergic or atopic backgrounds.^2^ Other important risk factors include structural abnormalities, esophageal dysmotility, gastroesophageal reflux disease, diabetes-related autonomic dysfunction, psychiatric illness, and behaviors such as rapid or excessive eating, especially during holidays and cultural events. Management typically involves urgent intervention because delays exceeding 24 hours significantly increase the risk of adverse events such as perforation. Flexible endoscopy is the cornerstone of treatment, offering both diagnostic and therapeutic capabilities through techniques such as gentle bolus advancement (“push”) or extraction with retrieval tools (“pull”).^3^ Although pharmacologic agents such as glucagon and effervescent drinks are sometimes used as temporizing measures, their success rates are inconsistent, and they should never delay definitive endoscopic management. Timely diagnosis and intervention remain key to preventing adverse outcomes.^4^

## Epidemiology

Esophageal food and foreign body (FB) impaction is a globally recognized concern, frequently seen in both pediatric and adult populations. FB ingestion and impaction, particularly in the esophagus, are common occurrences with more than 100,000 cases occurring each year.^1^ The prevalence varies by age group, with around 80% of cases occurring in children with peak incidence between age of 6 months and 6 years.^5^

Population-based data reveal gender and ethnic disparities, as well as age patterns, among those affected. In an epidemiologic study from Olmsted County, Minnesota, United States, the incidence of EoE increased significantly over the last few decades (from 0.35 [95% confidence interval {CI}], 0-0.87] per 100,000 person-years during 1991-1995 to 9.45 [95% CI, 7.13-11.77] per 100,000 person-years during 2001-2005), reaching 55.0 (95% CI, 42.7-67.2) per 100,000 persons as of January 1, 2006.^6^ Another epidemiologic study in California (USA) showed that the estimated annual incidence rate of episodes was 13 per 100,000, and the male:female ratio was 1.7:1.^7^ In a systematic review from 2007, 13 studies reported detailed data on 323 adult patients, with a mean age of 38 years (range: 14-89 years), of whom 76% were males.^9^ Another large retrospective study involving 548 patients with EFBI found that 59% were male and 68% were White, with a bimodal age distribution. Of these patients, 88% (482 individuals) required a procedure, and 63% (347 individuals) had food impactions.^10^

In addition to baseline risk, certain time periods, such as holidays and national events, show a measurable increase in impaction cases. Adults who underwent emergent esophagogastroduodenoscopy during cultural holidays or national athletic events were significantly more likely to present with esophageal food impaction than those treated during nonevent periods (36.8% vs 3.6%; *P* < .001).^11^ Meat has been consistently identified as the most common cause of food bolus impaction, accounting for approximately 85% of all cases.^8^

## Risk factors

A range of anatomic, physiologic, behavioral, and systemic factors contribute to the risk of esophageal impaction. Intrinsic factors such as anatomical malformations (eg, esophageal diverticulum, rings, and webs), dysmotility (eg, achalasia), and mucosal inflammation as a result of exposure to gastric refluxate as observed in gastroesophageal reflux disease are known risk factors for EFBI.^12^

The increase in prevalence of EFBI over the last 15 years has been associated with an increased prevalence of EoE and peptic-related strictures.^2^ In a prospective analysis of 261 patients presenting with dysphagia who underwent esophageal biopsies, EoE was pathologically diagnosed in 12% of all patients, and 35% of patients <50 years of age, with a relative risk of 9.5 if age <50 years.^13^ In a study by Heerasing et al^14^ involving 96 patients with food bolus impaction, 51 underwent biopsy. Among those biopsied, 37% had reflux esophagitis, 33% had EoE, 10% had Barrett’s esophagus, and 2% had achalasia. For the patients who were not biopsied, most showed endoscopic evidence of reflux esophagitis.^14^ Multiple other studies showed that EoE constitutes approximately 50% of patients with EFBI who underwent biopsy.^10^ Allergic and atopic disorders are closely linked to EoE and are therefore important contributors to impaction risk. EoE was present in 36.8% of patients with self-reported food allergies versus 9.3% of those without allergy (*P* < .001)^13^ as well as in 22% of asthmatics versus 9% of nonasthmatics (*P* < .01).^9^

In diabetic populations, autonomic neuropathy appears to play a significant role in esophageal dysmotility and subsequent bolus stasis. In a multicenter study, a gastric emptying study was performed in 84 patients with diabetes mellitus, of whom esophageal dysmotility was present in 63% of patients, and gastroparesis in 13%, independent of gender, symptoms, and type of diabetes. Longer duration of diabetes, however, correlated significantly with esophageal dysmotility.^15^ Esophageal motor disorders were identified in 46% of patients with diabetes, with diabetic retinopathy and the use of insulin recognized as predictive of esophageal motor abnormalities.^16^

Behavioral, psychiatric, and neurologic factors must also be considered in certain cases. In adults, nonfood FB impaction risk factors include psychiatric disorders, developmental delays, and alcohol intoxication.^17^ Moreover, events associated with rapid or excessive eating further increase the likelihood of bolus impaction. Patients with intrinsic risk factors should be advised to modify their diet during cultural events associated with tachyphagia and large meals to prevent esophageal food impaction.^11^

## Management

The majority of foreign objects—more than 80%—will pass through the gastrointestinal tract without the need for intervention.^18^ However, esophageal foreign objects and food impactions should be removed within 24 hours. Delaying removal lowers the chances of successful retrieval and increases the risk of adverse events, including perforation.^19^ A detailed approach to management of patients presenting with esophageal impaction is shown (Fig. 1).

**Figure 1:**
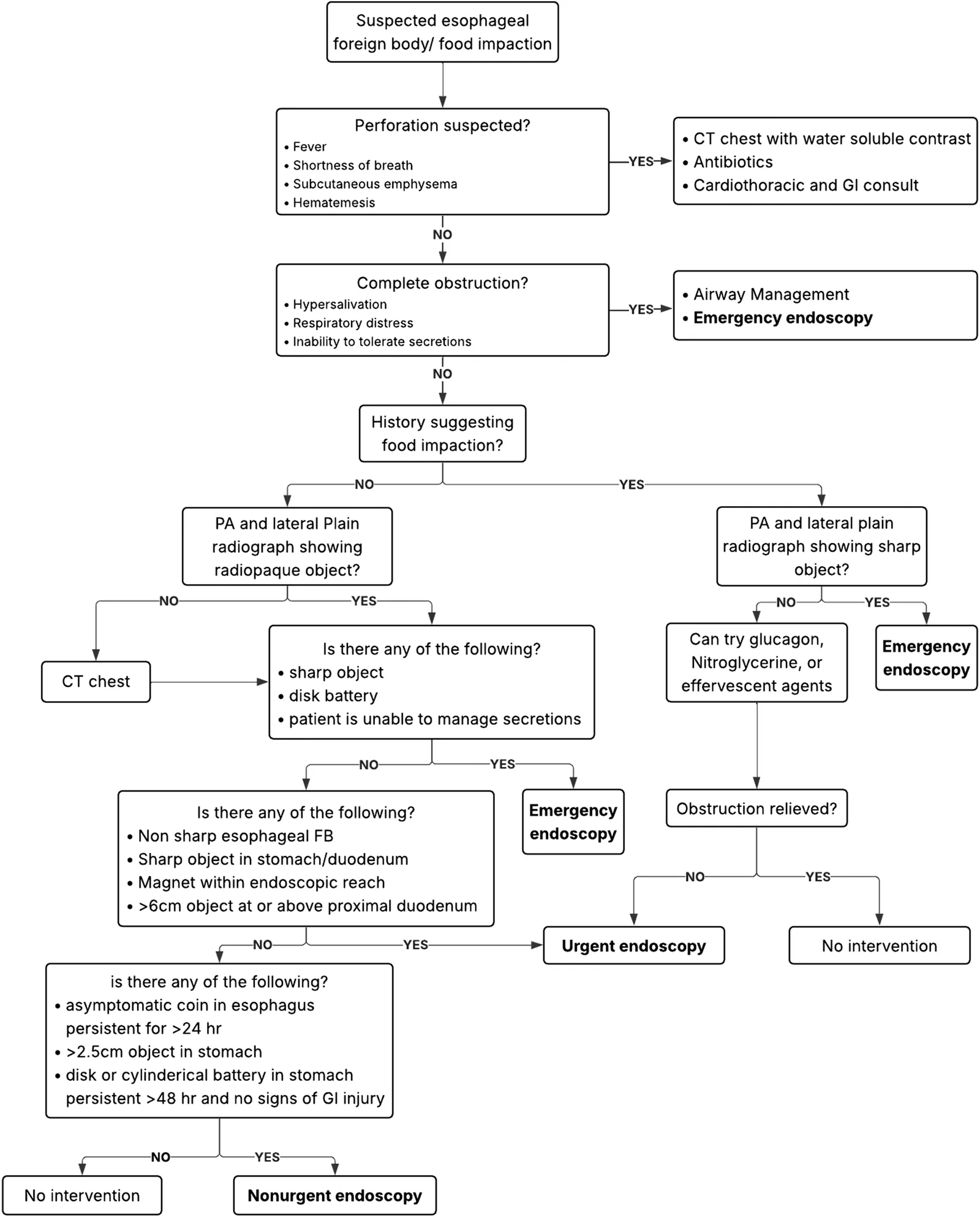
Approach to management of patients presenting with acute esophageal impaction. Emergency endoscopy: within 2 to 6 hours of patients presenting to health care facility. Urgent endoscopy: within 24 hours of presentation. Nonurgent endoscopy: 24 or more hours after presentation. *FB*, Foreign body; *GI*, gastrointestinal; *PA*, postroanterior.

In most cases, esophageal food bolus impaction can be managed effectively using flexible endoscopy. Commonly, this involves either gently pushing the bolus into the stomach or extracting it with endoscopic retrieval tools as discussed in the following:

The push technique, which requires advancing the endoscope to the site and applying gentle pressure to dislodge the bolus into the stomach, is generally safe when performed by experienced clinicians. However, it is not advised in cases involving sharp objects or when perforation is suspected.^3^ A combination of glucagon at the time of endoscopy can cause esophageal relaxation, which aids the success of the push technique.^20^ In a retrospective analysis from 2001, the push technique resolved the food impaction in 184 of 189 patients (97%) presenting with acute esophageal food impaction, confirming the safety and efficacy of the push technique in esophageal food impaction. The study also suggested that esophageal dilation at the time of disimpaction can be considered if there are no obvious contraindications.^21^

Alternatively, the “pull technique” uses various endoscopic tools to retrieve the food bolus. This method may be associated with mucosal trauma and can be time-consuming if multiple passes are required, in which case the use of an overtube may offer added protection.^3^

The timing of presentation and the site of impaction can significantly influence the risk of adverse events. Multivariate analysis revealed that a presentation delay of more than 2 days (*P* < .001), FB seen on lateral cervical radiography (*P* < .001), and impaction at the cricopharyngeus (*P* = .009) or upper esophagus (*P* = .005) were all significant independent risk factors for developing adverse events after FB ingestion.^22^

### Endotracheal intubation

Endotracheal intubation might be required emergently if the patient is unable to protect their airway because of any reason, or urgently in patients at high risk of aspiration (such as those with proximal esophageal location or a known full stomach), or who have significant secretions that cannot be managed otherwise, even in the absence of overt airway compromise.

Elective intubation is also often considered, particularly in cases where the procedure may be prolonged or technically challenging, or when there is a high risk of aspiration during the intervention.^23^ To our knowledge, there is limited high-quality evidence on the impact of elective intubation on adverse events, and some studies suggest higher adverse event rates in intubated patients, likely reflecting greater underlying risk rather than the intubation itself.^24^

The World Society of Emergency Surgery and the American Society for Gastrointestinal Endoscopy (ASGE) recommend that airway assessment should be individualized on the basis of patient factors, procedural risk, and local practice patterns; routine intubation is not universally required for all cases.^25^

### Pharmacologic and conservative management

Conservative management for esophageal soft food bolus impaction includes observation for spontaneous passage and pharmacologic interventions such as glucagon, effervescent agents, and other smooth muscle relaxants. However, the current consensus is that these approaches have limited efficacy and should not delay definitive endoscopic intervention. The most robust evidence, including a Cochrane systematic review, indicates that there are inadequate data to recommend any enteral or parenteral treatments, including glucagon, benzodiazepines, or effervescent agents, as reliably effective for disimpaction, and the certainty of evidence is low.^4^ These medications should be considered adjuncts or temporizing measures while awaiting endoscopy, when endoscopy is not readily available, or when it is declined by the patient.^26^

#### Glucagon

Glucagon is the most commonly used pharmacologic agent. Glucagon has inhibitory effects on the alimentary tract contractions and can significantly lower the lower esophageal sphincter pressure through interference with intramural cholinergic neuronal transmission.^27^ In a large retrospective study, glucagon was successful in 39.5% of cases, but endoscopic removal was nearly universally effective.^28^ However, a recent multicenter randomized trial found no difference between glucagon and placebo in resolving impaction or shortening procedure time^29^; in another study, it was found to be less effective in patients with underlying EoE.^30^ In a case series, a combination of glucagon at the time of endoscopy to cause esophageal relaxation, together with air insufflation and gentle pressure with the tip of the endoscope, can achieve easy passage of the food boluses into the stomach.^20^

#### Effervescent agents

Effervescent agents (eg, carbonated beverages) have shown some promise in retrospective studies, with higher success rates than glucagon, but the evidence is limited and not definitive. In a retrospective study including 239 patients with acute esophageal food impaction, effervescent agent monotherapy showed 55.6% success rate, compared with 17.7% with glucagon monotherapy, and 17.2% in those who had no therapy.^31^

#### Benzodiazepine

A randomized controlled trial included in a Cochrane review evaluated intravenous diazepam (10 mg) followed by glucagon versus placebo and found no significant difference in disimpaction rates (38% vs 32%; risk ratio, 1.19; 95% CI, 0.51-2.75), with low certainty of evidence and no clear benefit over placebo.^4^

#### Nitrate

Similarly, a prospective single-arm pilot study of oral nitroglycerin solution (0.4 mg, up to 3 doses) found symptom resolution in only 11.8% of subjects, a rate comparable to placebo and other pharmacologic agents, and noted that several patients could not tolerate the intervention because of inability to swallow or headache.^32^

#### Topical swallowed steroids

A small case series evaluated budesonide orodispersible tablets in patients with suspected EoE and food impaction, with 38% (3/8) experiencing resolution without endoscopy, but this is insufficient to support routine use.^33^

### Endoscopic management

#### Indications and timing of endoscopic intervention

A retrospective study found that the sooner endoscopy was performed, the more likely it was to successfully locate the esophageal FB. In patients in whom localization was successful, the mean ± standard deviation time to scope was 289.75 ± 465.94 minutes, compared with 471.06 ± 659.93 minutes in those in whom localization failed (*P* = .028), where time to scope is defined as the time interval from when patients visited the emergency department or an outpatient clinic or during hospitalization to the time when the endoscopy procedure was done:^34^

According to the ASGE, the indications for endoscopic management of FB ingestion are categorized on the basis of the urgency of the situation and summarized as follows^35^:

Emergent endoscopy, which should be done within 2 to 6 hours of presentation to medical care,^25^ is required for patients unable to manage their secretions because of esophageal obstruction, those with disk batteries, or sharp-pointed objects in the esophagus, because these pose a high risk of perforation or other severe adverse events.

Urgent endoscopy, within the first 24 hours of presentation,^36^ is indicated for nonsharp EFBI, esophageal food impactions without complete obstruction, sharp objects in the stomach or duodenum, long objects in the proximal duodenum, and magnets within endoscopic reach.

Nonurgent endoscopy, which can be performed >24 hours of presentation, is advised for coins in the esophagus (which may be observed for 12-24 hours in asymptomatic patients), objects in the stomach with diameter >2.5 cm, and disk batteries in the stomach if there are no signs of gastrointestinal injury, with removal recommended if the battery remains in the stomach beyond 48 hours.

#### Retrieval tools

##### Through-the-scope biliary extraction balloon

In 2015, Anand et al^37^ introduced the use of the through-the-scope (TTS) biliary extraction balloon (Fig. 2A) as a method for retrieval of EFBI or esophageal food impaction when traditional methods, such as retrieval net, polypectomy snare, or rat-tooth forceps, fail. The TTS balloon can effectively retrieve nonsharp objects by passing the deflated balloon distal to the foreign object, inflating it, then pulling it back in settings where other retrieval tools are not effective. When visualization is obscured by the FB, a guidewire can be used to guide the instrument and allow safe advancement of the balloon.

**Figure 2:**
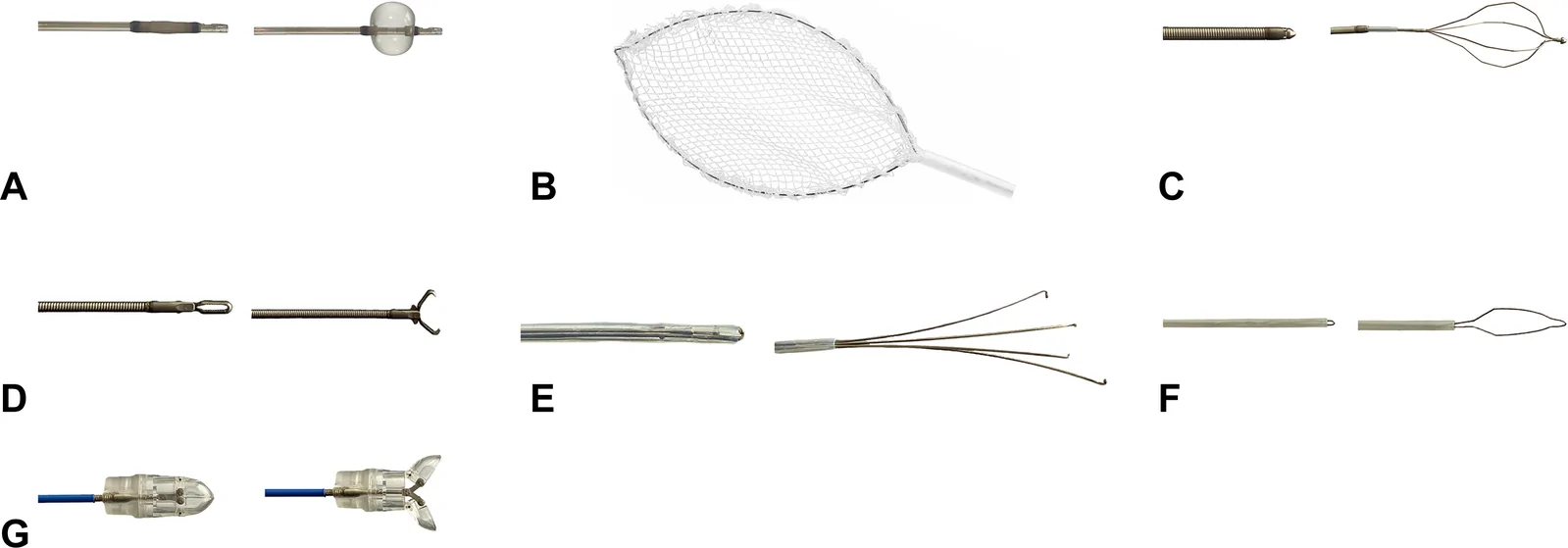
Endoscopic tools: (**A**) through-the-scope biliary extraction balloon; (**B**) retrieval net; (**C**) retrieval basket; (**D**) grasping forceps; (**E**) Talon (US Endoscopy Group Inc/STERIS Corporation, Mentor, Ohio, USA) grasping device; (**F**) polypectomy snare; (**G**) over-the-scope grasper.

##### Retrieval net

The retrieval net (Fig. 2B) is indicated for removal of soft, amorphous food boluses and blunt FBs. It is deployed through the working channel, envelops the material, and allows for en bloc extraction. Advantages include high efficacy for food bolus removal, minimal trauma, and the ability to retrieve large or friable material; disadvantages are limited utility for sharp or hard objects and potential difficulty with very large or impacted boluses. The retrieval net is specifically highlighted as highly effective and safe for food bolus and button battery retrieval.^38^

##### Retrieval basket

The retrieval basket (Fig. 2C) is used for small, round, or smooth FBs and occasionally for a fragmented food bolus. It is advanced beyond the object, opened, and then withdrawn to ensnare the item. Advantages are secure grasping of small objects; disadvantages include limited efficacy for large, irregular, or soft boluses and risk of mucosal trauma if excessive force is applied.^39^^,^^40^

##### Grasping forceps

Grasping forceps (Fig. 2D) (eg, rat-tooth, alligator, and optical forceps) are indicated for firm, irregular, or sharp-pointed objects. They are advanced enough to directly grasp the object. Advantages are precise control and a strong grip for sharp or metallic items; disadvantages are the risk of mucosal injury and limited efficacy for large or soft boluses. It should not be used for button batteries. In children, optical forceps are preferred for blunt objects, and rat-tooth forceps for sharp items.^39^^,^^41^

##### Talon grasping device

The Talon (US Endoscopy Group Inc/STERIS Corporation, Mentor, Ohio, USA) grasping device (Fig. 2E) is a specialized forceps with multiple prongs for improved grip on irregular or slippery objects. It is used similarly to standard forceps but offers enhanced grasping strength. Advantages are improved security for difficult objects; disadvantages are increased device complexity and potential for trauma.^42^

##### Polypectomy snare

The polypectomy snare (Fig. 2F) is used for looped or elongated objects and for fragmenting large food boluses. The snare is looped around the object and tightened. Advantages include versatility and ability to fragment; disadvantages are the risk of mucosal laceration and limited efficacy for amorphous boluses.^39^

##### Over-the-scope grasper

The over-the-scope grasper (Fig. 2G) is designed for large, tenacious, or adherent food bolus, blood clots, or necrotic debris. They attach to the endoscope tip and provide a large secure grasp. They are highly effective for large or adherent material and easy to use, but are limited by availability and lack of extensive comparative data.^43^

#### Esophageal dilation

A large retrospective cohort study found that immediate esophageal dilation after disimpaction was infrequently performed (18% of cases) but was associated with a low adverse event rate and reduced risk of early recurrence compared with delayed or no dilation. No adverse events were reported in the immediate dilation group, supporting its safety in experienced hands.^44^ According to the American College of Gastroenterology clinical guidelines, esophageal dilation is an effective treatment and was one of the first therapies used for adult patients with EoE. In these patients, if a critical stricture is encountered or food impaction has occurred, then dilation can be performed during initial endoscopy.^45^ However, in patients with suspected malignant strictures or strictures resulting from caustic ingestion, esophageal dilatation is associated with a higher risk of perforation and bleeding, warranting careful risk-benefit assessment.^24^

The ASGE notes that the risk of bleeding and perforation with esophageal dilation is very low, and that the choice between balloon and bougie dilators does not significantly affect adverse event rates.^25^

##### Balloon dilator

Case series have demonstrated that using TTS and over-the-wire balloon dilators (Fig. 3A) is a rapid, safe, and effective method for relieving food impaction, with high success rates and minimal adverse events, although these data are limited to small studies. Balloons up to 20 mm are typically used for benign strictures, whereas larger balloons (30-40 mm) are reserved for specific indications such as achalasia.^46^

**Figure 3:**
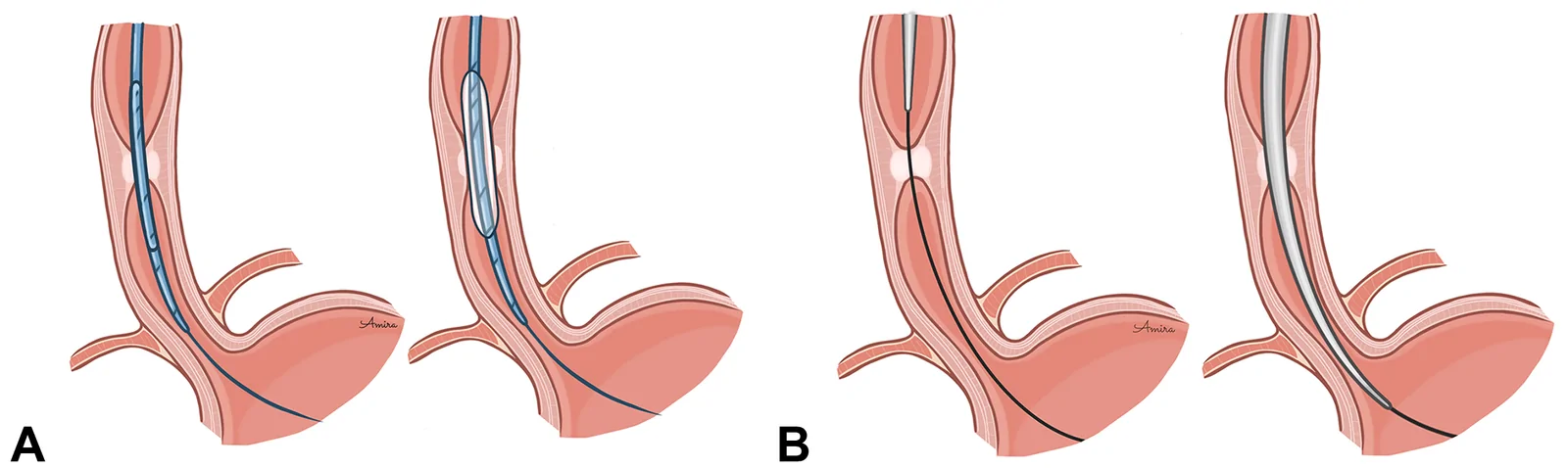
Devices used for esophageal dilation: (**A**) esophageal balloon dilator; (**B**) Savary-Gilliard bougie dilator (Cook Medical, Bloomington, Ind, USA).

The World Society of Emergency Surgery recommends that if a large food bolus is jammed in the lower esophagus and cannot be managed with push or retrieval techniques, gentle passage of a balloon catheter past the obstruction, followed by inflation and withdrawal, can be used to disimpact the bolus, which may then be retrieved with a net; however, it emphasizes that these are adjunctive techniques and not first-line approaches.^25^

##### Bougie dilator

These dilators include weighted push-type (Maloney) and polyvinyl wire–guided (Savary-Gilliard; Cook Medical, Bloomington, Ind, USA) dilators (Fig. 3B). Maloney dilators are passed blindly or under fluoroscopic guidance and do not require a guidewire but are not suitable for complex or very narrow strictures because of the risk of buckling and perforation. Savary-Gilliard dilators are more rigid, used over a guidewire, and allow for controlled, graded dilation. Both types are available in a range of sizes (16-60F).^47^

#### Adjunctive tools

##### Overtube

Overtubes can be used to protect the airway and facilitate endoscope passage during the removal of multiple or sharp objects. It may facilitate repeated esophageal intubations with minimal trauma, especially for piecemeal removal of incohesive or large food boluses. However, their use is less common in pediatric patients because of the risk of esophageal injury, especially in patients who have esophageal stricture or have previously undergone head and neck surgery or radiation therapy.^48^

Overtubes have 1 diameter that fits the scope diameter of 9.8 to 11.7 mm, but 2 different lengths: 50 cm (Fig. 4A) and 25 cm (Fig. 4B). The 25-cm overtube is usually used for foreign objects impacted in the esophagus, and the 50-cm overtube for sharp objects distal to the esophagus. Using the 50-cm overtube for esophageal sharp FBs might be inappropriate because it can bypass the FB, causing more mucosal injury.

**Figure 4:**
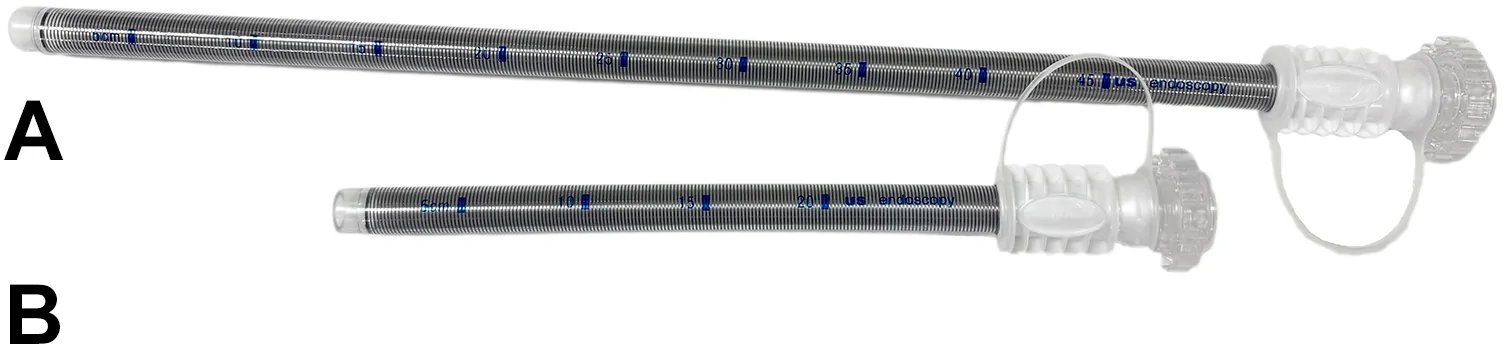
Overtube: (**A**) length 50 cm; (**B**) length 25 cm.

##### Endoscopic cap

Endoscopic caps (Fig. 5) are transparent, rigid, plastic attachments fitted onto the distal tip of the endoscope. Cap-assisted techniques have been shown in randomized controlled trials to significantly reduce procedure time, increase technical success, improve en bloc removal rates, and decrease mucosal injury compared with conventional techniques without a cap. The cap creates a chamber at the tip, allowing the endoscopist to trap and extract the bolus, including food bolus or sharp FBs, more efficiently with less risk of mucosal injury.^49^

**Figure 5:**
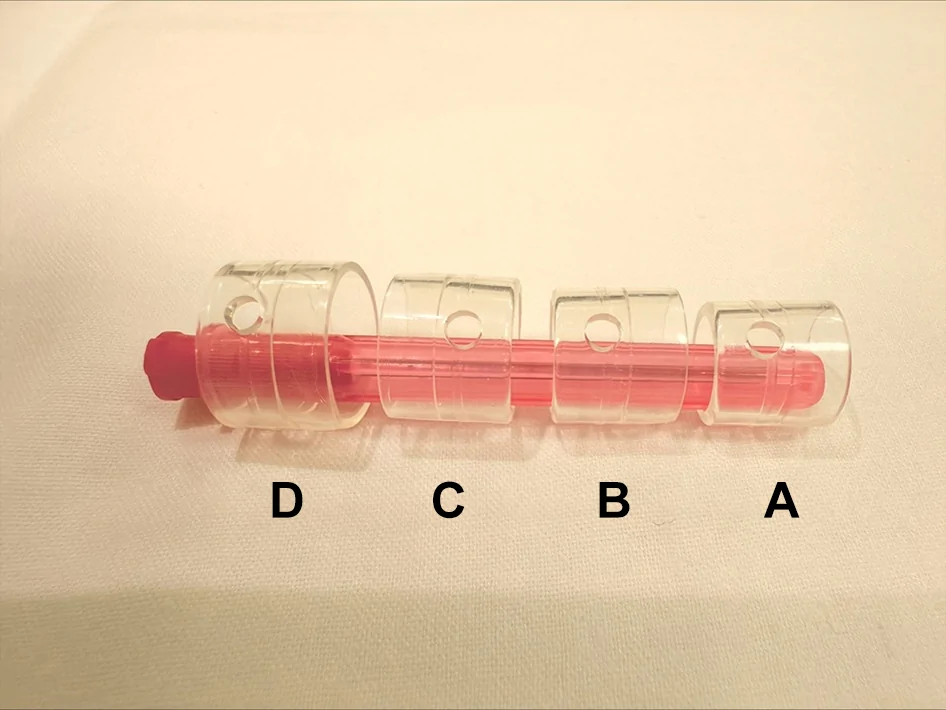
Endoscopic caps: (**A**) outer diameter 11.35 mm, fits scopes 8.9 to 9.5 mm; (**B**) outer diameter 12.4 mm, fits scopes 9.8 to 10.5 mm; (**C**) outer diameter 13.4 mm, fits scopes 10.9 to 11.8 mm; (**D**) outer diameter 15 mm, fits scopes 12.6 to 13.2 mm.

##### Endoscopic hood

Endoscopic hoods (Fig. 6) are described in the medical literature as effective adjuncts for the safe removal of sharp objects during endoscopic procedures. These devices are designed to shield the mucosa from direct contact with sharp or pointed FBs during extraction, thereby reducing the risk of laceration or perforation. It can successfully enclose sharp or pointed objects and prevent mucosal injury during removal from the upper gastrointestinal tract, especially the stomach.^50^A summary of endoscopic tools, their indications, advantages, and disadvantages is provided in Table 1.

**Figure 6:**
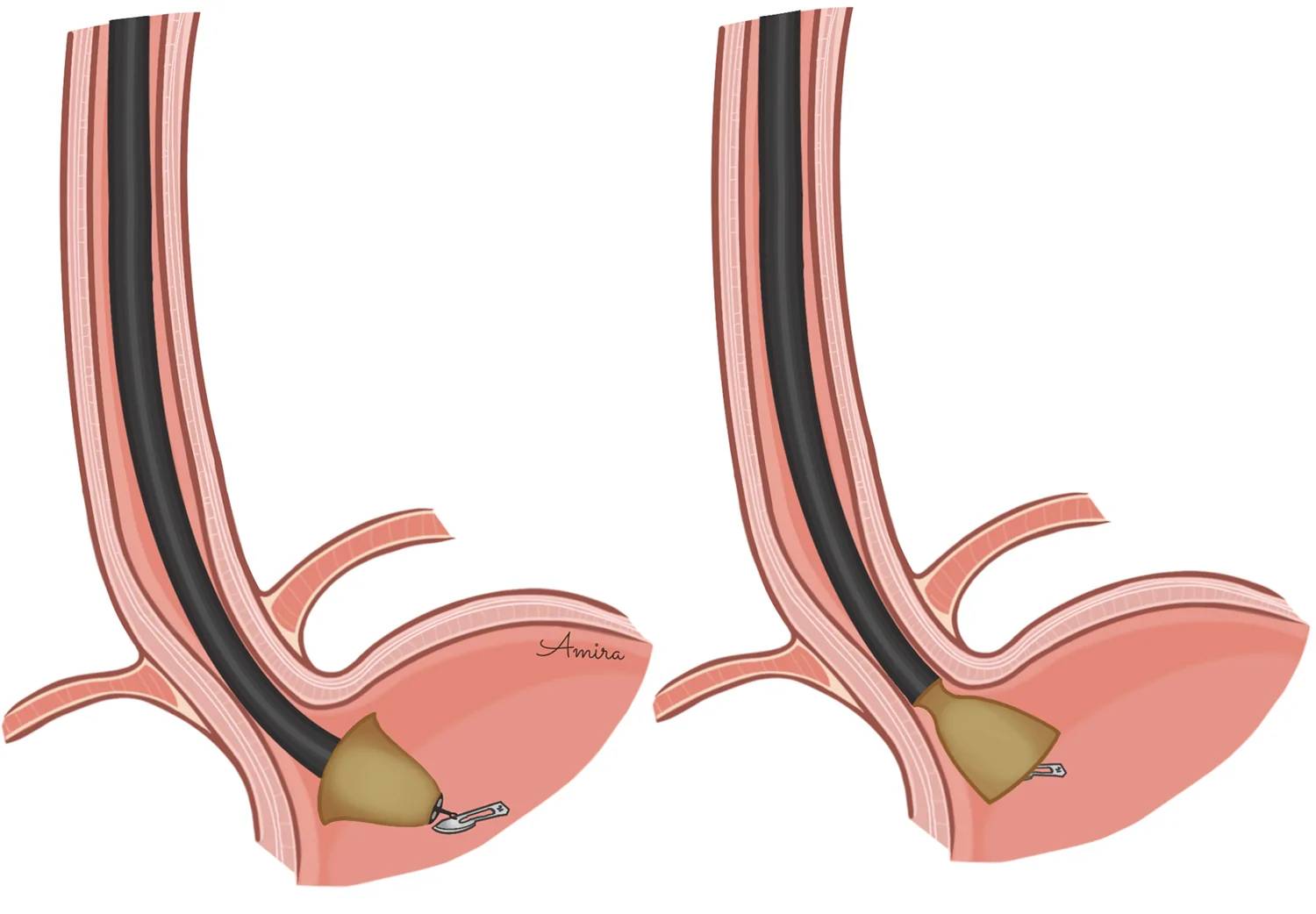
Endoscopic hood.

#### Esophageal biopsy

In a prospective analysis of 261 patients presenting with dysphagia, of which 31 patients had biopsy-proven EoE, 13/31 (42%) did not have the classic esophagogastroduodenoscopy findings (rings and/or furrows) and would have been missed without esophageal biopsies.^13^

The ASGE emphasizes that esophageal biopsy sampling should be performed even in the absence of typical signs of EoE, such as rings, exudates, or furrows, if there is a clinical suspicion of the disease. The sensitivity of histopathology increases with the increase in the number of biopsy samples taken; the sensitivity of 2, 3, and 6 biopsy samples was 84%, 97%, and 100%, respectively. Biopsies should be deferred only in rare cases where patient instability or high aspiration risk precludes safe sampling.^51^

In this video (Video 1, available online at www.igiejournal.org), we show multiple retrieval tools being used in a simulation of impacted food or foreign objects in the esophagus.

## Conclusions

EFBI and esophageal food impaction are urgent conditions where timely endoscopic intervention is critical. Flexible endoscopy is the reference standard and should not be delayed by pharmacologic measures such as glucagon, which have limited efficacy. The ideal approach is guided by object type and location.

**Table 1:** Endoscopic tools used in esophageal food/FB impaction

Technique/tool	Indication of use	Advantages	Disadvantages
Through-the-scope balloon (Fig. 2A)	Disimpaction of large FBs or food bolus, especially when standard tools fail	Can push or pull; rapid and effective; minimal adverse events	Risk of mucosal trauma; limited data
Retrieval net (Fig. 2B)	Removal of soft, amorphous food bolus and blunt foreign bodies	Minimizes fragmentation; high efficacy for food and button battery	Limited for large/firm objects; may require multiple passes; not suitable for sharp items
Retrieval basket (Fig. 2C)	Small, round, smooth FBs or fragmented food bolus	Secure grasp of small/round items.	Risk of mucosal trauma if forceful; less effective for large or soft boluses
Grasping forceps (Fig. 2D)	Firm, irregular, or sharp-pointed objects	Precise control; strong grip for sharp or metallic items	Risk of mucosal injury; limited efficacy for soft or large boluses
Talon (US Endoscopy Group Inc/STERIS Corporation, Mentor, Ohio, USA) grasping device (Fig. 2E)	Irregular or slippery foreign bodies	Improved grip strength	Increased complexity; potential for trauma
Polypectomy snare (Fig. 2E)	Looped or elongated FBs; fragmentation of large food boluses	Adjustable loop; versatile; can fragment bolus	Risk of laceration; less effective for soft or amorphous bolus
Over-the-scope grasper (Fig. 2F)	Large, tenacious, or adherent food bolus, blood clots, necrotic debris	Large, secure grasp; easy to use; effective for bulky material	Limited availability; lacks comparative outcome data
Balloon dilator (Fig. 3A)	Postdisimpaction esophageal dilation or for stricture-related impaction	Rapid, safe, effective; minimal adverse events	Requires experience; large balloons (30-40 mm) for specific cases
Bougie dilator (Fig. 3B)	Benign esophageal strictures	Graded dilation; range of sizes; Savary-Gilliard offers controlled guidance	Risk of perforation with Maloney; not ideal for complex, narrow strictures
Overtube (Fig. 4)	Piecemeal removal, multiple passes, sharp object removal, airway protection	Protects the airway; minimizes trauma with repeated intubation	Bulky; risk of injury; less used in pediatric/postradiation patients
Endoscopic cap (Fig. 5)	Soft bolus or sharp FBs; improves visualization, en bloc extraction	Shorter procedure; improves success; reduces mucosal injury; acts as a barrier	May not fit larger FBs; requires a compatible endoscope
Endoscopic hood (Fig. 6)	Sharp-pointed FBs; shields mucosa during removal	Provides safe enclosure of sharp objects; reduces risk of injury	Limited to case series; dependent on device availability

*FB*, Foreign body.

### For food bolus impaction

The push technique is highly effective, or a retrieval net can be used for extraction. Cap-assisted removal reduces procedure time and mucosal injury. If a stricture is found, immediate esophageal dilation is safe and reduces recurrence risk.

### For sharp or pointed objects

Emergent endoscopy (within 2-6 hours) is required. Consider endotracheal intubation for airway protection. Use grasping forceps with an overtube (25 cm) or an endoscopic hood to prevent laceration during withdrawal.

### For blunt, nonfood objects

Select tools based on shape: a retrieval basket for small round objects; a polypectomy snare for elongated items; or forceps. A TTS balloon can be effective when other tools fail.

In summary, successful management requires prompt endoscopy, the use of protective adjuncts, appropriate tool selection, and taking esophageal biopsy samples for evaluation for underlying pathology such as EoE or strictures to prevent recurrence.

## Patient consent

Patient consent was not required as the authors used simulated videos without any patient contact using prosthetic/synthetic materials.

## Disclosure

The following author disclosed financial relationships: H. Siddiki: Consultant for Boston Scientific and Conmed. All other authors disclosed no financial relationships.

## Supplementary data

Video 1 In vitro simulation for endoscopic approaches and instruments used for esophageal foreign body and impacted food retrieval.
